# Interdisciplinary and Participatory Research for the Elimination of Socially Determined Diseases in the Amazon: A Narrative Review

**DOI:** 10.1590/0037-8682-0487-2025

**Published:** 2026-07-17

**Authors:** Alícia Patrine Cacau dos Santos, Vinícius Azevedo Machado, Wuelton Marcelo Monteiro, Felipe Leão Gomes Murta

**Affiliations:** 1 Universidade do Estado do Amazonas (UEA), Escola de Ciências da Saúde, Manaus, AM, Brasil.; 2 Fundação de Medicina Tropical Doutor Heitor Vieira Dourado (FMT-HVD), Departamento de Ensino e Pesquisa, Manaus, AM, Brazil.

**Keywords:** Health inequities, Socially determined diseases, Community-Based participatory research, Qualitative research, Amazon region

## Abstract

This narrative review examines how interdisciplinary and participatory research has advanced the understanding and control of socially determined diseases in the Brazilian Amazon. The integration of social sciences into the Tropical Medicine Foundation Dr. Heitor Vieira Dourado marks a shift from purely biomedical approaches toward those recognizing the cultural, environmental and territorial dimensions of health. Qualitative studies on snakebite envenoming among Indigenous populations revealed that treatment integrates biomedical and spiritual practices, highlighting the need for intercultural dialogue. In malaria elimination, research emphasized evaluating the acceptability and feasibility of new tools such as point-of-care G6PD testing and single-dose tafenoquine, demonstrating that understanding perceptions of innovations is indispensable for effective implementation. Studies on human immunodeficiency virus (HIV) prevention and trachoma among Indigenous and urban populations illustrated how stigma, gender relations and structural inequalities shape health outcomes. Extreme climate events, such as the 2023 Amazon drought, exacerbate vulnerabilities by disrupting livelihoods and healthcare access. The article concludes by proposing an interdisciplinary research agenda aligned with Brazil's Healthy Brazil Program, emphasizing community engagement, intersectional analysis, One Health integration and implementation science as key strategies for equitable disease elimination in the Amazon.

## BUILDING AN INTERDISCIPLINARY PERSPECTIVE IN TROPICAL MEDICINE

The Brazilian Amazon is home to over 31 million people distributed across vast and diverse territories^1^. Many live in traditional riverine and Indigenous communities that depend on forests and waterways for their livelihoods. Yet, persistent poverty, geographic isolation and limited access to primary healthcare services continue to shape profound health inequalities^2^. Between 2023 and 2025, more than 130,000 riverine families reported per capita incomes below half the minimum wage, and nearly 117,000 were living in extreme poverty^3^. These conditions intersect with environmental degradation and deforestation, reinforcing cycles of vulnerability and limiting access to adequate sanitation, clean water and timely emergency care^4^.

Top-down health interventions have often failed to achieve sustainable outcomes because they overlook local knowledge systems, cultural logics and territorial barriers^5^. In the Amazon, addressing these inequities requires more than biomedical innovation; it demands approaches that recognize the coexistence of distinct epistemologies and health systems, foster intercultural dialogue and engage communities as equal partners in the design and implementation of health actions^6^. In this sense, community-based participatory research (CBPR) has emerged as a transformative alternative that bridges science and social practice through dialogue and collective action. By positioning communities as active partners rather than passive subjects, CBPR encourages shared decision-making, contextual understanding and community-driven efforts for solutions. This interdisciplinary perspective reflects a necessary shift toward research that is not only about people but conducted with them and for them^7^.

## INTERDISCIPLINARY APPROACHES TO HEALTH RESEARCH IN THE AMAZON

This article presents a narrative review synthesized from a corpus of qualitative studies conducted by our interdisciplinary research group (LIPeSQ/FMT-HVD). The studies included in this review were selected based on two main criteria: (1) they had to be qualitative investigations addressing socially determined diseases or health vulnerabilities in the Amazon region, and (2) they had to employ participatory or community-based methodologies involving Indigenous, riverine, or other vulnerable populations. The corpus encompasses original studies published in peer-reviewed journals, covering snakebite envenoming, malaria elimination, HIV/AIDS prevention, trachoma, and climate-related health impacts. Rather than providing a systematic synthesis, this narrative review aims to integrate key lessons learned across these studies to propose a consolidated, interdisciplinary research agenda aligned with Brazil's Healthy Brazil Program.

The establishment of the Interdisciplinary Laboratory of Social and Qualitative Research (LIPeSQ) within the Tropical Medicine Foundation Dr. Heitor Vieira Dourado (FMT-HVD) represents a turning point in how tropical diseases are studied and understood in the Brazilian Amazon. While FMT-HVD has long been recognized for its biomedical excellence in diagnosis, treatment and molecular surveillance of infectious diseases, the integration of social and qualitative research brings a complementary perspective that foregrounds the lived experiences, cultural meanings and territorial realities of affected populations. This approach acknowledges that health and disease in the Amazon are not only biological phenomena but also deeply social and environmental processes.

The following sections illustrate how interdisciplinary and participatory methodologies have been applied to address complex health problems in Amazon. Each case demonstrates that understanding disease in its social, cultural and environmental context is important for developing effective and equitable health interventions. 

### Snakebite envenoming (SBE)

SBE remains one of the most neglected and dramatic public health problems in the Brazilian Amazon. It exposes deep inequalities in healthcare access among riverine and Indigenous populations who rely on fishing, farming and forest-related activities for survival. Long distances, lack of transport and the concentration of antivenom availability in urban hospitals often delay treatment, resulting in otherwise preventable complications and deaths^8^ ^-^ ^10^.

Qualitative research conducted by our group with Tikuna, Kokama and Kambeba Indigenous caregivers revealed that SBEs are perceived not only as biological accidents but also as events with social and spiritual dimensions. Snakebites are linked to daily subsistence activities such as fishing or hunting, but they can also have spiritual causes, which are attributed to sorcery or social disharmony within the community. The latter are considered more complex, demanding both ritual healing and biomedical care to restore balance and promote recovery. Treatment therefore unfolds in multiple, complementary stages: immediate self-care, community-based herbal and ritual treatment, hospital care with antivenom, and post-discharge spiritual recovery. Indigenous caregivers recognize antivenom as essential but emphasize that care must also restore social and spiritual equilibrium^11^.

Health professionals who work exclusively with Indigenous people also share this understanding that SBE treatment should integrate traditional and biomedical practices^12^. Qualitative research conducted with 56 healthcare professionals revealed that most recognized the limits of strict biomedical care and emphasized the need for collaboration with traditional healers and community leaders. Participants described how the coexistence of both systems can enhance trust, improve treatment adherence and reduce delays in seeking care^13^. 

### Perceptions, Practices and Challenges in Malaria Elimination

Despite notable progress in decreasing malaria cases in the Brazilian Amazon over the past twenty years, complete elimination still demands considerable effort. Brazil’s National Malaria Elimination Plan aims to stop malaria transmission by 2035^14^. Reaching this goal involves not only deploying new malaria elimination tools but also understanding how health interventions are carried out and experienced in real-world settings. 

Treatment adherence is essential in the context of the elimination of malaria, as incomplete or interrupted therapy sustains transmission and increases the risk of relapse in *Plasmodium vivax* infections. Qualitative findings from our study showed that adherence is shaped by social, economic and health system factors that influence how patients experience care. Daily work routines, geographic mobility and the pressure to resume income-generating activities often interfere with treatment completion^15^. Interruptions were not viewed as neglect but as pragmatic responses to social constraints and to the side effects of primaquine, commonly interpreted as a sign of the drug’s potency. To address these challenges, mobile phone reminders were co-developed with patients and tested as a support tool. Participants valued the clarity and timing of short text messages, describing them as a simple yet meaningful link with the health service that helped them remember to take their doses and feel cared for^16^. 

Qualitative data from urban, peri-urban and rural settings across the Amazon indicate that malaria is often perceived as a routine part of life in the forest. This normalization weakens the perceived urgency of prevention and completion of treatment. Patients and health workers have described context-specific explanations for malaria transmission, about transmission, such as associations with contaminated water or food, and have expressed skepticism about the feasibility of elimination. Participants in community discussions emphasized that “malaria has always been here”, reflecting a sense of coexistence with the disease that complicates prevention efforts^15^. 

One of our studies with gold miners working on the borders of the Amazon adds further nuance to the social dimensions of elimination. Participants frequently describe stigmatization when returning to towns, where they were blamed for introducing malaria, and many expressed not understanding how the disease spreads. Self-medication practices were widespread, including the use of medicinal plants and drugs purchased in illegal markets, often leading to incomplete treatment. While miners generally trusted diagnosis and cure in principle, this trust was conditional and closely linked to accessibility, the perceived competence of providers, and previous experiences with health services. These accounts reveal how social stigma, mobility and precarious labor conditions intersect sustain malaria transmission in the Amazon^17^.

The recent introduction of tafenoquine and point-of-care quantitative G6PD testing in Brazil represents another critical step toward eliminating *P. vivax* ^18^. Yet, the success of these innovations depends on their acceptance and effective use in real-world settings. Qualitative evaluations involving healthcare professionals and patients revealed both optimism and apprehension. Health professionals viewed the new regimen as a significant advance that simplified supervision and improved adherence, but many requested continued training to gain confidence in interpreting G6PD thresholds and counseling patients^19^. Patients appreciated the convenience of a single dose and their rapid recovery but initially expressed doubts about its efficacy, which gradually diminished through direct experience and transparent communication. Field teams also noted that tafenoquine’s distinctive packaging enhanced credibility, although its bulk posed logistical challenges in remote areas. These experiences demonstrate that technological progress alone cannot ensure success. Achieving elimination requires integrating social insights into every stage of policy design and implementation^20^.

### Socially Determined Diseases and Intersecting Vulnerabilities in the Amazon

Profound inequalities in access to healthcare remain one of the most persistent obstacles to controlling and eliminating socially determined diseases in the Amazon. The HIV/AIDS epidemic in Manaus illustrates how structural and symbolic dimensions of inequality converge to limit prevention and care^21^. Despite the availability of effective biomedical tools such as antiretroviral therapy and pre-exposure prophylaxis (PrEP), access remains deeply stratified by geography, class, gender and stigma. Qualitative research among men who have sex with men revealed that many participants faced institutional neglect, moral judgment and fragmented information within health services. Long travel distances, limited clinic hours and a lack of culturally sensitive care led to recurrent interruptions in treatment and continuity in prevention. For some, seeking care implied exposure to discrimination and social labeling, leading many to delay or avoid seeking care at health facilities^22^. 

Evidence from the Tocantins region, where a qualitative study was conducted among the Krahô people, further demonstrates how structural determinants influence health outcomes and perceptions of illness caused by trachoma. Despite targeted interventions for trachoma elimination in the region, local understandings of disease etiology remained grounded in environmental and spiritual beliefs, with limited interaction between ancestral knowledge and Western scientific perspectives. Community members associate eye discomfort not with hygiene or infection, but with natural agents such as wind, dust and smoke from pollution. Participants also described recurrent diarrheal and skin diseases linked to polluted water and the lack of functional sanitation systems. In both participating villages, communal latrines and waste management systems were installed but never operated effectively, forcing residents to revert to open air defecation and bathing in the river. Gendered divisions of care also emerged as a key aspect of health governance. Women took primary responsibility for domestic health tasks like bathing children, preparing herbal remedies and maintaining hygiene. At the same time, men kept decision-making power over medical treatments and interactions with outside agents. This dynamic influenced their participation in health programs and acceptance of preventive measures. These findings highlight the limitations of standardized education campaigns that equate disease prevention with individual hygiene practices. In contexts where illness is socially and environmentally mediated, culturally grounded approaches are essential to bridge epistemological gaps and promote local agencies in health action (unpublished data).

Today, these longstanding inequities are being compounded by emerging environmental crises that disproportionately affect the most vulnerable populations. One of the defining challenges of our time is the convergence of social inequality, infectious diseases and environmental disruption. One example was the severe drought of 2023, which became one of the most critical ecological crises in recent history for communities along the Upper Solimões River. 

The extreme drop in river levels paralyzed fluvial transport, cut off access to food and healthcare, and exposed the dependence of Amazonian livelihoods on river dynamics. As recurrent floods followed the drought, these climate extremes have become a defining feature of life in many parts of the region, where environmental instability intersects with persistent social inequities. As demonstrated in previous research, extended droughts and subsequent floods profoundly affect riverine livelihoods, mobility, and access to essential services. When water levels drop, entire communities become isolated, blocking access to healthcare. During floods, families are displaced, schools shut down, and exposure to contaminated water increases the rate of diarrheal and vector-borne diseases. Women’s accounts emphasized the fragility of maternal and child health under these conditions, reporting childbirths along riverbanks and delayed emergency care due to the absence of transport and communication. These findings show how extreme climate events operate as amplifiers of inequality, producing cascading effects that extend beyond physical health to social and economic well-being^23^. The Table 1 shows research topics examined by our interdisciplinary laboratory and methodological approaches for deeper investigation.

**TABLE 1:** Research topics and qualitative approaches used in health research conducted by LIPeSQ/FMT-HVD.

Research topic	Qualitative methods	Population Territory	Main findings	Potential contribution
Vulnerable populations and access to health services	Participant observation;	Indigenous/Riverine	Riverine communities and indigenous perceive SBE and other illnesses as interconnected with their environment, daily subsistence activities, and limited access to healthcare.	Decentralization strategies in health services for communities in remote areas.
	In-depth Interviews.	Amazonas State		
Vulnerable populations in the Amazon border region and challenges for malaria elimination.	Participant observation;	Gold Miner	Malaria is part of routine occupational risk, but stigma, mobility, and self-medication practices undermine elimination efforts.	Development of contextually appropriate health communication strategies.
	In-depth Interviews;	Amapá State		
	Focus group discussion.			
Understanding treatment adherence and discontinuation for HIV/AIDS.	Field immersion;	LGBTQ+ community	Stigma, institutional neglect, fragmented information, and long travel distances are the main barriers to PrEP access among MSM in the Brazilian Amazon.	Development of strategies for reducing stigma in healthcare services.
	Community dialogue circles;	Amazonas State		
	Photovoice			
Understanding the structural determinants of trachoma.	Field observation;	Indigenous	Trachoma as a symptom of historical territorial neglect, persisting due to lack of basic sanitation, environmental contamination, and culturally inappropriate interventions.	Development of educational programs that integrate scientific and traditional knowledge.
	Focus group discussion; Community assemblies.	Tocantins State		
Understanding the impacts of environmental degradation on the lives of riverine people.	Participant observation;	Riverine	Climate extremes disrupt riverine communities' access to healthcare, food, and transportation, intensifying existing social and gender vulnerabilities.	Development of healthcare protocols for extreme climate scenarios.
	In-depth Interviews;	Amazonas State		
	Focus group discussion.			

**AIDS:** Acquired Immunodeficiency Syndrome; **HIV:** Human Immunodeficiency Virus; **LGBTQI+ -:** Lesbian; Gay: Bisexual, Transgender, Queer and other identities; **MSM:** Men who have sex with Men; **PrEP:** Pre-Exposure Prophylaxis; **SBE:** Snakebite Envenomation.

The studies discussed reflect the specific geographic, institutional and historical context of western Brazilian Amazon, and may not be directly transferable to other regions with different social, ecological or healthcare configurations. Despite these constraints, presenting this consolidated trajectory offers valuable insights into the process of building interdisciplinary capacity and the lessons learned through sustained community engagement. The proposed research agenda below is intentionally open and designed to stimulate collaboration with other research groups and institutions working toward the common goal of eliminating socially determined diseases in the region.

## PROPOSED RESEARCH AGENDA FOR STRENGTHENING INTERDISCIPLINARY RESEARCH IN THE ELIMINATION OF SOCIALLY DETERMINED DISEASES IN THE AMAZON

The launch of the Healthy Brazil Program *(Programa Brasil Saudável)* in 2024 marks a milestone in national public health policy, bringing together 14 ministries in a multisectoral effort to eliminate eleven socially determined diseases and five vertically transmitted infections by 2030^24^. This initiative reframes disease elimination as a collective social project rather than a purely clinical mission. It recognizes that health equity depends on integrated actions in housing, education, environment, Indigenous rights and social protection. Within this framework, the Amazon region occupies a critical position. It embodies the intersection of geographic isolation, cultural diversity and environmental change, factors that both challenge and enrich the implementation of national policies. 

The Healthy Brazil Program creates an opportunity to strengthen this agenda by linking community engagement with multisectoral governance^24^. Implementation science offers the tools to evaluate what works, for whom and under what conditions, while frameworks such as the Consolidated Framework for Implementation Research (CFIR) guide the integration of evidence, context and collaboration^25^. 

To advance this goal, a research agenda for the coming years could prioritize several complementary lines of inquiry:

- Monitor and evaluate implementation processes to understand how local adaptations, political shifts and institutional learning affect the sustainability of health interventions in diverse Amazonian contexts.
- Expand research on digital inclusion and health communication as critical strategies to reduce geographic isolation. Participatory studies that explore how Indigenous and riverine populations appropriate or resist digital technologies can inform culturally sensitive models of communication, surveillance and health education.
- Address intersectional and gendered dimensions of health equity by examining how gender, age and ethnicity intersect to shape vulnerabilities and access to care.
- Engage decision-makers and public managers to ensure that evidence informs real-time policy adjustments.
- Integrate qualitative research within One Health frameworks to better understand how environmental degradation, zoonotic risks and community livelihoods interact.
- Expand postgraduate training to promote master’s and doctoral research on interdisciplinary topics in Tropical Medicine.

## FINAL REMARKS

Achieving the 2030 elimination goals, consistent with the United Nations Sustainable Development Goals and Brazil’s Healthy Brazil Program, will require sustained investment and collective action across multiple levels of governance and sectors of society. Federal, state and municipal governments must work in conjunction with scientists from diverse areas of research and civil society to ensure that resources, knowledge and decision-making are shared equitably. Strengthening Amazon-based institutions and supporting local researchers to lead qualitative and participatory studies will not only enhance scientific autonomy but also increase the relevance and legitimacy of research outcomes. The path toward eliminating socially determined diseases in Brazil, and particularly in the Amazon, depends on merging biomedical innovation with social, cultural and territorial understanding. 

## Data Availability

Research data is available in the body of article.
